# Classification of Non-Conventional Ships Using a Neural Bag-Of-Words Mechanism

**DOI:** 10.3390/s20061608

**Published:** 2020-03-13

**Authors:** Dawid Polap, Marta Wlodarczyk-Sielicka

**Affiliations:** 1 Marine Technology Ltd., 81-521 Gdynia, Poland; d.polap@marinetechnology.pl; 2 Department of Navigation, Maritime University of Szczecin, Waly Chrobrego 1-2, 70-500 Szczecin, Poland

**Keywords:** bag-of-words mechanism, machine learning, image analysis, ship classification, marine system, river monitoring system, feature extraction

## Abstract

The existing methods for monitoring vessels are mainly based on radar and automatic identification systems. Additional sensors that are used include video cameras. Such systems feature cameras that capture images and software that analyzes the selected video frames. Methods for the classification of non-conventional vessels are not widely known. These methods, based on image samples, can be considered difficult. This paper is intended to show an alternative way to approach image classification problems; not by classifying the entire input data, but smaller parts. The described solution is based on splitting the image of a ship into smaller parts and classifying them into vectors that can be identified as features using a convolutional neural network (CNN). This idea is a representation of a bag-of-words mechanism, where created feature vectors might be called words, and by using them a solution can assign images a specific class. As part of the experiment, the authors performed two tests. In the first, two classes were analyzed and the results obtained show great potential for application. In the second, the authors used much larger sets of images belonging to five vessel types. The proposed method indeed improved the results of classic approaches by 5%. The paper shows an alternative approach for the classification of non-conventional vessels to increase accuracy.

## 1. Introduction

Ship classification is an important process in practical applications in different places. In coastal cities, ships enter from the mouth of a river or moor at ports. This type of activity is quite often reported and recorded. However, for measurement, statistical, or even analytical purposes, it is often necessary to record vessels that arrive but do not report anywhere. To this end, the simplest solution is to create a monitoring system and analyze acquired images. This type of system architecture is based primarily on three main components: video recording, image processing, and classifying possible water vehicles.

While the solution itself seems simple, each component has its disadvantages, which also affect the others. First, the video recorder may be a simple camera, but often one needs to take good-quality photos for easier analysis. The second component is image processing. Image processing should consider the location of a possible ship on an image, or even perform some extraction of features. It is particularly important to remove unnecessary areas such as the background, houses, and even water. The third element is classifying these images, i.e., based on the obtained images, the algorithm should determine with some probability the type of ship.

In this paper, we considered the third aspect of such a system to model a solution enabling the most accurate classification of a given type of ship based on a photo entered into the system. In the analyzed system [1] an important element was the recording of information about passing vessels in water bodies. Unfortunately, this task is not easy due to the similarities between ships and the many factors that can be mistaken for a ship.

## 2. Related Works

In the last decade, the number of methods for classifying images has increased, and the main contribution is the description of convolutional neural networks (CNNs). These mathematical structures can be modeled for specific classification problems, as can be seen in [2]. The classification problem might also be improved by extracting some important objects. For this purpose, segmentation can be used [3,4]. In this paper, the authors proposed a convolutional network architecture based on three dimensions of the incoming image. Moreover, CNN was used for classifying objects from different points of view, which is very practical using drones [5], or even for sleep stage scoring based on electroencephalogram (EEG) signals [6]. Moreover, CNN has been used for recognition of vehicle driving behavior [7]. In addition to classic architectures, there are others, such as U-net, which are used as segmenting elements and in inverse problems [8]. Many applications of CNN can be found, primarily in monitoring systems and medicine. 

In general, a database for training such structures has the biggest impact on the classifier. A very common problem is the lack of enough samples, which results in low efficiency or even overfitting. Data augmentation, i.e., generating new samples based on existing samples using image processing techniques, is a popular solution [9]. In [10], the authors discuss the effect of augmented data on CNN based on images from chest radiographs. Similarly, in [11], the authors use augmentation to increase the dataset by adding some distortions, such as changing the brightness, rotating, or adding some mirroring effects. Moreover, in recent years learning transfer has been enabled, i.e., the use of trained network architectures to minimize training time. The main idea was to create architectures and train them on huge databases. Trained classifiers are those whose coefficients are specialized in searching for features and classifying, so the learning transfer consists of using the finished model, modifying only selected layers, and overtraining only selected values to meet the needs of new bases. Not modifying any of the layers is called freezing. One of the first architectures for that was AlexNet [12]. Another was VGG16, modeled by a group from Oxford who primarily reduced the size of the filter in a convolution layer [13]. Another popular model is Inception [14], which drastically reduced the number of architecture parameters.

The ship classification problem depends on using images. Commonly used are synthetic aperture radar (SAR) images, by which ships can be classified based on their shape [15]. Similar research was described in [16,17], where superstructure scattering features were analyzed in the process of classification. Similarly, in [18], the idea of ship classification was solved by analyzing sound signals and removing the background sound of the sea. Other input data are aerial images that present a top view of the scenery and the ship. In all of these solutions, CNN was used for faster feature extraction and classification. An interesting approach was presented in [19], where the authors described the impact of simulated data on the training process of neural classifiers in the problem of ship classification. Moreover, in [20], a neural approach for ship type analysis with sea traffic was presented as an automatic identification system. All these studies used neural classifiers for image processing and classification.

In this paper, we propose a solution for the classification of different non-conventional ships using images made from the side and not from the top, like SAR images. This problem is hard, because images can be created using different light, from a different distance, or even from different sides of the object. The described solution was based on splitting the image of the ship into smaller parts (using keypoint algorithms with clustering) and classifying them by CNN into vectors that can be identified as features. This idea is a representation of a bag-of-words mechanism, where created feature vectors might be called words, and using these words, a solution can assign them a specific class. The main contribution of this article is the use of a bag-of-words mechanism to classify non-conventional ships, which in the future could be used in an innovative system for automatic recognition and identification in video surveillance areas. The solution proposed in the paper has not been applied anywhere and is a new approach to the subject.

## 3. Bag-Of-Words

Bag-of-words is an abstract model used in the processing of text or graphics. It is the representation of data described in words, i.e., linguistic values. In the case of two-dimensional images, with a word we can describe a feature or fragment of an object. The idea of using a bag can help classify processes, because the input image will be decomposed into smaller fragments and classified according to certain linguistic values. These values can help in the classification of larger objects. This is especially desirable when analyzing the same objects that differ in their small features.

The proposed idea consists of extracting small fragments of the image with certain features. All points are divided against a certain metric into smaller images containing a fragment of the object. Such images can represent everything, because the object can be on any background; for instance, a ship can be captured in the port or against a background of trees; in that case, smaller images can even show some trees. Thus, the use of a classical approach, that is, the creation of a bag-of-words using an algorithm such as k-nearest neighbors, is not very effective. The reason is the lack of connection between the features (in smaller parts), because it should be considered that the objects can be on different scales or turned at a certain angle or even have some noise, such as bad weather or additional objects. That is why we propose a bag-of-words model based on more complex structures, such as neural networks. 

### 3.1. Feature Extraction

The main idea of this study was to extract features using one of the classic algorithms for obtaining keypoints, such as scale-invariant feature transform (SIFT) [21], speeded up robust features (SURF) [22], features from accelerated segment test (FAST) [23], or binary robust invariant scalable keypoints (BRISK) [24], and then create samples with found features. It should be noted that if these algorithms processed the original image, the found points would probably cover the entire image; in the case of a simple image where a ship is at sea, all points could be placed on this object or water or waves, but there may be an image with some additional background with many possible points. To remedy this, in the first step, the image must be processed, which means using graphic filters to minimize elements such as edges or points. We used only two filters, such as gamma correction and blur.

### 3.2. Feature Extraction Based on Keypoints

Using the described algorithms, we obtained a set of keypoints, which we can describe as *A = {(x*_0_,*y*_0_*)*, *(x*_1_,*y*_1_*)*, *…*, *(x_n−1_*,*y_n−1_)}.* To minimize the number of points (because unnecessary elements of the image can be indicated), all points were checked against their neighbors. If the point had a neighbor within a certain distance α, it remained in the set. Otherwise, the point was removed, and the cardinality was reduced by one. The distance between two points *p_i_ = (x_i_*, *y_i_)* and *p_j_ = (x_j_*, *y_j_)* was checked using one of the two classic metrics, Euclidean or river. The best known is the Euclidean, modeled as

(1) $$d_{E}=\left(\left(x_{i},\;y_{i}\right),\left(x_{j},\;y_{j}\right)\right)=\sqrt{{\left(y_{i}-x_{i}\right)}^{2}+{\left(y_{j}-x_{j}\right)}^{2}}$$

A river metric is the distance between points but counted relative to a certain straight line between the points. For both points, a perpendicular projection is made, as a result of which an additional two points are obtained, *(x_o_*, *y_o_)* and *(x_p_*, *y_p_)*. The distance in this metric will be calculated as the sum of the distance of a given point to the straight line, the distance between these two points on the straight line, and the transition from the straight line to the second point. Formally, it can be stated as

(2) $$d_{R\;}\;\left(\left(x_{i},\;y_{i}\right),\left(x_{j},\;y_{j}\right)\right)=d_{E}\left(\left(x_{i},\;y_{i}\right),\left(x_{o},\;y_{o}\right)\right)+d_{E}\left(\left(x_{o},\;y_{o}\right),\left(x_{p},\;y_{p}\right)\right)+d_{E}\left(\left(x_{p},\;y_{p}\right),\left(x_{j},\;y_{j}\right)\right)$$

Depending on the given metric, all points are checked to see if the distance is smaller, and if so, the point is removed. The next step is to divide the points into subsets *B_q_*, where *q* is the number of objects. It is not possible to adjust the value of *q* without empirically checking and testing the data in the database. With this value, it is worth using existing algorithms to divide these points (for example, using the k-nearest neighbors algorithm). However, this value is unknown, so another approach to the topic should be taken. For this purpose, one of the previously described metrics can be used.

For all points in a given set *A*, the average distance value is calculated as

(3) $$\xi \left(A\right)=\frac{1}{5\;\cdot \;n^{2}\;}\;\overset{n-1}{\underset{i=0}{\sum }}\overset{n-1}{\underset{j=i}{\sum }}d_{metric}\left(\left(x_{i},\;y_{i}\right),\left(x_{j},\;y_{j}\right)\right)\;$$

With the average distance, the points are divided concerning this value. The first subset is created by adding the first point to it, i.e., $\left(x_{0},\;y_{0}\right)\;\in \;B_{0}$. Then, for each point $\left(x_{r},\;y_{r}\right)\;\in \;A$, we check to see if the distance between this point and any other in a given subset $\left(x_{0},\;y_{0}\right)\;\in \;B_{0}$ is less than the average distance of the set, i.e.,

(4) $$d_{metric}\left(\left(x_{r},\;y_{r}\right),\left(x_{0},\;y_{0}\right)\right)<\;\xi \left(A\right)\;$$

If the above equality is met for a point $\left(x_{r},\;y_{r}\right)$, it is added to subset $B_{0}$ and removed from A. In the case where none of the points is added to a given subset, another subset, $B_{1}$, is created. Then, the first point from A is added to subset $B_{1}$ and removed from A. In this way, the action is repeated to meet the stop condition, which is the emptiness of the set, A=∅.

**Figure 1:**
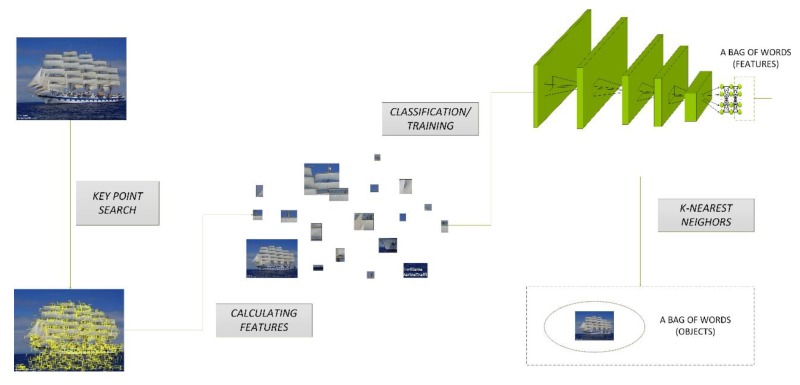
Graphic visualization of the proposed model.

As a result, subsets B are generated, with each representing one feature. For each set, an image is created whose dimensions will depend on the subset. To find the dimensions, we look for the maximum and minimum values of both coordinates in a subset that we can mark as *x_max_*, *y_max_*, *x_min_*, and *y_min_*. Hence the image size will be *(x_max_–x_min_) × (y_max_–y_min_).* Then, the images are saved and each one represents a part of the image. The left part of Figure 1 shows this process of extracting smaller parts of the image. Figure 1 shows a graphic visualization of the proposed model.

## 4. Classification with Bag-Of-Words

Unfortunately, there was no unambiguous method to assign attributes to specific groups automatically. Therefore, we suggested creating groups at the initial stage of modeling the solution with the help of empirical division. In this way, the basic database of features were created, which will include a later bag-of-words.

### 4.1. Convolutional Neural Network

One of the most important branches of artificial intelligence methods is neural networks, which have been modeled for the needs of graphic image classification. Convolutional neural networks are models inspired by image processing by the cerebral cortex of cats. It is a mathematical structure built of three types of layers, where the layers between them are connected by synapses burdened with a certain weight. The weight is given randomly while creating the structure. Then, in the training process, the weights are modified to best match the training database.

One of the key layers of the network is the convolutional layer, which takes the image of the input with dimensions *w × h × d*, where *w* and *h* are the width and height of the image and *d* is understood as depth and depends on the model. For color images saved in the red–green–blue (RGB) model, the depth will be 3 due to the number of components. Formally, each image is saved as a set of matrices, each of which describes the image values for a given component. The convolutional layer works on a principle of image filter *f* of size *k × k*. This filter is a matrix with *k*^2^ coefficient defined randomly and modified during the training process. This filter is moved over the image and changes the value in pixel *p* on image *I* at position *(i*, *j)*, which can be defined as

(5) $$I\left[i,\;j\right]=\frac{1}{K}\;\overset{\left[\frac{k}{2}\right]}{\underset{t=-\left[\frac{k}{2}\right]}{\sum }}\overset{\left[\frac{k}{2}\right]}{\underset{r=-\left[\frac{k}{2}\right]}{\sum }}I\left[i+t,\;j+r\right]\cdot f\left[t,\;r\right]$$

where matrix *f* is located over an image and the central point of the matrix is over a pixel at position *(i*, *j)*, and *K* is the sum of all weights of filter *f*. The main purpose of this layer is feature extraction and reduction of data redundancy on the image. Applying some filter on the image will change it; depending on the coefficient of filters, some objects might be deleted or highlighted.

The second type of layer is called pooling, which has only one purpose: to reduce the size of matrices. Reducing depends on some function *g(∙)*, which selects one pixel from each square *m × m*. The most commonly used function is *max(∙)* or *min(∙).*

These two layers can be used alternately many times. In the end, there is the last layer, the fully connected layer, which is understood as a classical neural network. Each pixel from the last layer (pooling or convolutional) is input as a numerical value. This layer is composed of columns of neurons connected by synapses, which are burdened with some weight. Each neuron gets a numerical value that is processed and sent to the next column. This operation can be described as

(6) $$x_{m}^{t}=f(\overset{n}{\underset{i=1}{\sum }}x_{i}^{t-1}\;\cdot \;\omega_{i})\;$$

where *x^t^* is the output from neuron *m* in layer *t*, and $\omega_{i}$ is a weight on the connection between *x_m_* in layer *t* and *x_i_* in layer *t* – 1. The number of columns and neurons depends on the modeled architecture. In the last column, there should be *k* neurons (when a classification process is described as a *k*-classes problem). The final calculation of an image in such a structure gives a probability distribution that can be normalized by some function like softmax. These values are understood as the probability of belonging to this class.

Unfortunately, all weights in this model are generated randomly at the beginning. To change these values, the training algorithm must be used. The main idea is to minimize loss function during two iterations. One such algorithm commonly used in convolutional networks is adaptive moment estimation [25]. The modification of weights is based on a basic statistical coefficient like the correlation of mean $\hat{m}$ or variation $\hat{v}$:

(7) $$\hat{m_{t}}=\frac{m_{t}}{1-\beta_{1}^{t}}$$

(8) $$\hat{v_{t}}=\frac{v_{t}}{1-\beta_{2}^{t}}$$

where *m_t_* and *v_t_* are the mean and variation values in the *t*th iteration. The formulas for calculating this can be presented as

(9) $$m_{t}=\beta_{1}m_{t-1}+\left(1-\beta_{1}\right)g_{t}$$

(10) $$v_{t}=\beta_{2}v_{t-1}+\left(1-\beta_{2}\right)g_{t}^{2}$$

where $\beta_{1},\;\beta_{2}$ are distribution values.

Those two statistical coefficients are used in the modification of weight as

(11) $$\theta_{t+1}=\theta_{t}-\frac{\eta }{\sqrt{{\hat{v}}_{t}+\epsilon }}\;\hat{m_{t}}$$

where η is the learning coefficient and ϵ ≠0, which prevents division by 0.

### 4.2. Bag-Of-Words

A trained classifier can be used as an element dividing incoming images into selected elements in a bag. For each image, smaller images representing features are created. Each of these features is classified using the pretrained convolutional neural network. As a result, the network will return the probability of belonging for each word in the set (each single output from the network is interpreted as a word). Based on a certain probability and features, it is possible to assign these attributes to an object. The selection of features for an object works on the principle of determining conditional affiliation to another word in the bag. To make it impossible to save the whole object to its characteristics, it is worth introducing division of the bag into two sets (or even two bags). The first bag will contain only features and the second full objects. For a better understanding of this idea, let us assume that the image presents a motorboat. The biggest bag will contain a class of ships, like motorboat, yacht, etc. The smallest bag (in the biggest one) will describe one ship. For motorboat, these words would be, for example “a man”, “waves”, and “no sails”.

Each of these objects is defined as a numerical vector consisting of zeros and ones (ones as belonging to this class). Each item in the vector is assigned to one feature from the bag-of-words, so its creation consists of using the result returned by the classifier. It should be noted that for many smaller segments from basic images, there will be many classification results. These results are averaged by all returned decision from classifiers.

The evaluation of the feature vector to an object occurs by comparing these vectors. The simplest method is to approximate the values returned by the network to integers and compare them with the words in a bag. However, there may be a situation where the vector will be different in one position compared to the patterns. To prevent this, we suggest using the k-nearest neighbors algorithm, which will allow assigning to a given object. The full display of this process is shown in Figure 1.

The *k*-nearest neighbors algorithm consists of analyzing and assigning the sample to neighboring samples [26,27]. Suppose that the value *x_i_* has an assigned class *μ_i_.* In the case of the analyzed problem, *x_i_* will correspond to 1 and values of *μ_i_* are the appropriate values representing the objects. The algorithm finds the nearest neighbors (values) $x_{n}^{\prime }\;\epsilon \;\left\{x_{0},\;x_{1},\;\ldots ,\;x_{n-1}\right\}$ for the given value *x* according to the following equation:

(12) $$\min\left(d_{metric}\;\left(x_{i},\;x\right)\right)=d_{metric}\;\left(x_{n}^{\prime },\;x\right),\;\;\;i=0,\;1,\;2,\;\ldots ,\;n-1.$$

## 5. Experiments 

In our experiments, we tested two databases. The first one had two classes, sailing ship and others, and was used to create the first set of features and find the best combination of algorithms. The second database contained more classes and the biggest number of samples to show the potential application of such an approach.

### 5.1. Classification for Two Classes of Ships

In these experiments, we tested the proposed solution to find the best combination for our proposition. For this purpose, the database we used was very small. It contained two classes, sailing ship and others. A sailing ship should have sails, although they do not always have to be spread. Such an observation allows the creation of two features describing this object, i.e., masts and sails. In this way, a vector describing these two classes will be created:

(13) $$\{\begin{array}{c} \left[1,\;1\right]\;\;\;\;\;\;\;\;\;\;sailing\;ship\\ \left[0,\;0\right]\;\;\;\;\;\;\;\;\;\;\;\;\;\;\;\;other \end{array}$$

where individual values are understood as appropriate features, masts and sails. In these tests, a CNN architecture as described in Table 1 was used. 

In the experiments, we used a database contained 800 images (600 with sailing ships, and 200 with other ships). In the training process, 75% of the samples selected randomly from each class were used, and the remaining 25% was used for the validation process, which were 150 and 50 images.

For each sample, one of the keypoint algorithms was used, which allowed us to create a few smaller segments. We tested the algorithm for each segment, and the results of two selected metrics, Euclidean and river, are presented in Table 2. In the table, for each algorithm, there are two columns labeled “Object features” and “Background”, which means that the extracted segment describes an important feature of a ship or not. Quite a common problem was to find the background, i.e., an insignificant fragment of the ship, and a large amount of sky or sea. The results shown are averaged over the entire base. It is easy to see that using the Euclidean metric generates many more features compared to the river metric. In both cases the ratio of images depicting features of the background exceeded 50%; however, that is not that big for the classic Euclidean metric.

**Figure 2:**
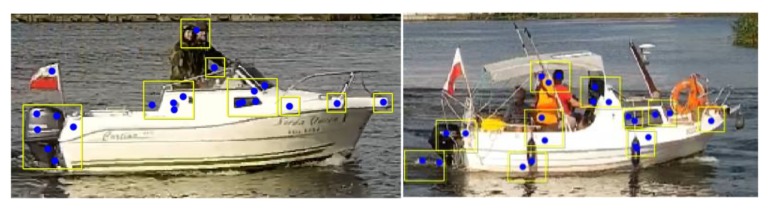
Examples of clustered keypoints for motorboat images.

In our tests, we used the SIFT, SURF, BRISK, and FAST algorithms to find keypoints. After that, all found segments were resized to one size and calculated using CNN. The results obtained for each image were averaged and classified using the k-nearest neighbors algorithm (in this experiment, k = 2) and are presented in Table 3 and Table 4 (the results in the second column represent classification of the whole image). Some examples of keypoint clustering are presented in Figure 2.

The highest efficiency was obtained with the Euclidean metric using the SURF algorithm. For this combination, the results of classification compared to those without using the bag-of-words mechanism was nearly 6% higher than that with the convolutional network alone. However, it is worth noting that the significant difference between the results obtained indicates the negative predictive value, whose value was almost twice as high when using the bag mechanism. This factor determines the probability of assigning a false sample to the correct class; in this case, not a sailing ship. The situation is similar to other hybrids, where this value is always higher than 50%. A similar situation occurred with the F1 score, which is the harmonic average of the precision and recall coefficients. This factor allows us to evaluate the classification if its components have different values. In each case, the statistical coefficients indicated a more accurate process taking into account the proposed mechanism.

**Figure 3:**
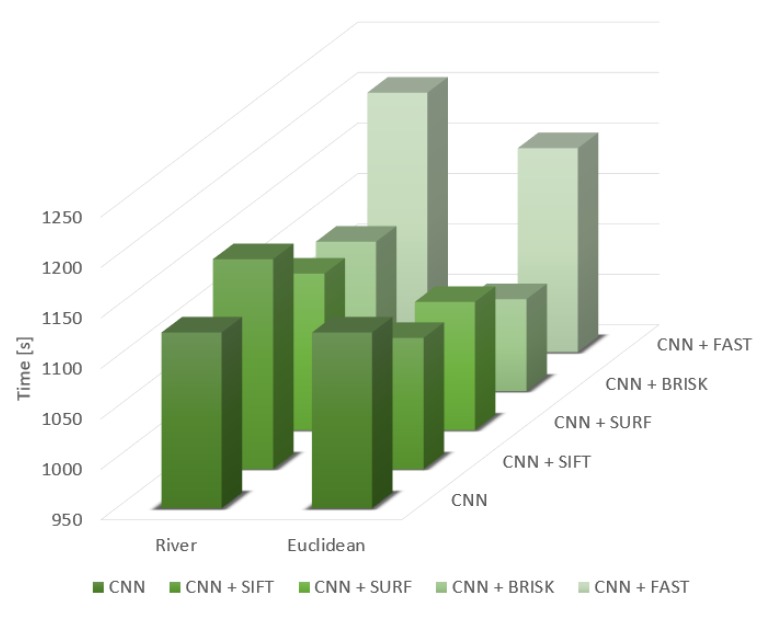
Average time for image processing and the training process.

For a more detailed analysis, time measurements were also made for the image processing and training of a given architecture, as shown in Figure 3. 

The presented results are averaged data from 10 tests. In general, using the Euclidean metric saves approximately 10% more time than using the river metric. The tests showed that the longest processing time occurred using the FAST algorithm and the shortest with BRISK. As for the SIFT and SURF algorithms, the time measurement was at a similar level and was classified as in the middle.

### 5.2. Classification for Five Classes of Ships

Based on the previous results, the best accuracy was achieved with a combination of the SURF algorithm and CNN. We used this combination for classification of five classes: cargo (2120 images), military (1167 images), tanker (1217 images), yacht (688 images), and motorboat (512 images). For the first three classes, images were downloaded from a publicly available dataset from Deep Learning Hackathon organized by Analytics Vidhya. Each class was divided randomly into two sets in a 75%:25% (training/validation) ratio, and for the training process the data were split in the same proportion. Using the training set, the SURF algorithm was used to create smaller parts, and based on the created sets, these samples were put into features which can be described as the following vector:

(14) [mast, sail, people, color, simplyShape],

where *people* means that on deck some people can be found, *color* means that a boat can have different colors (for a military ship, it is mainly gray), and *simplyShape* means that the ship can be recognized as a simple geometric figure, such as a rectangle. These features were chosen according to the database used and their possible location.

Using these features, words describing ship type were defined as follows:

(15) $$\{\begin{array}{c} \left[0;\;0;\;0;\;1;\;1\right]\;\;\;\;\;\;\;\;\;\;cargo\\ \left[1;\;0;\;0;\;0;\;0\right]\;\;\;\;\;\;\;\;military\\ \left[0;\;0;\;0;\;1;\;0\right]\;\;\;\;\;\;\;\;\;tanker\\ \left[1;\;1;\;1;\;1;\;0\right]\;\;\;\;\;\;\;\;\;\;yacht\\ \left[0;\;0;\;1;\;1;\;1\right]\;\;\;\;\;motorboat \end{array}$$

The training database contained 4278 images, which resulted in almost 26,000 smaller segments. Data were split into features based on color clustering using the *k*-means algorithm [28] and corrected empirically (especially for shape). We trained the classifiers with the architecture described in Table 1, but in the end there were five because of the five classes of features. The classifier was trained for five different numbers of iterations, t∈{20, 40, …, 100}, and the accuracy is presented in Table 5. The best accuracy was reached using 80 iterations; accuracy did not improve with more iterations.

The obtained accuracy is not very promising in such a classification problem. The main cause of this is the selection of features and creating sets for them. In the experiments, the dataset was so big that whether the sample belonged to the set was determined by the algorithm. Moreover, a feature such as shape is not the best choice for ships.

Despite these drawbacks, we conducted an additional experiment to check the classification result for this database in terms of hybrids. We classically trained a CNN to classify full images. Next, we checked the effectiveness of the validation base. Then we combined the obtained results from this classification with the proposed solution. Our approach classified into a given class out of the bag, so we understand the assignment to this class as adding a constant value equal to 0.2 to the probability of assignment according to the classic classifier. This approach will allow one probability distribution to be changed by 20%. The results of such action are shown in Table 6. The table shows the exact numbers of correctly classified images from the validation set and the accuracy.

These data show that our proposition can be used as an additional component and increase the classification accuracy by nearly 5.5%. This result is better, but there is a problem with more time to train nets and classify samples because of much more operation. It is worth noting that values increased mainly for the military class, yachts, and motorboats. This is due to the good definitions of features such as the ones we have for military, or people and colors for the other two classes. The main conclusion is that the most difficult task is to initially declare a bag-of-words describing these features. This solution can be used in practice, but there are some additional tasks during the modeling of this solution, such as overseeing the creation of small images representing features and assigning them to individual groups. Also, the declaration of characteristics involves allocating image segments to these classes and analyzing them before training the classifier.

**Figure 4:**
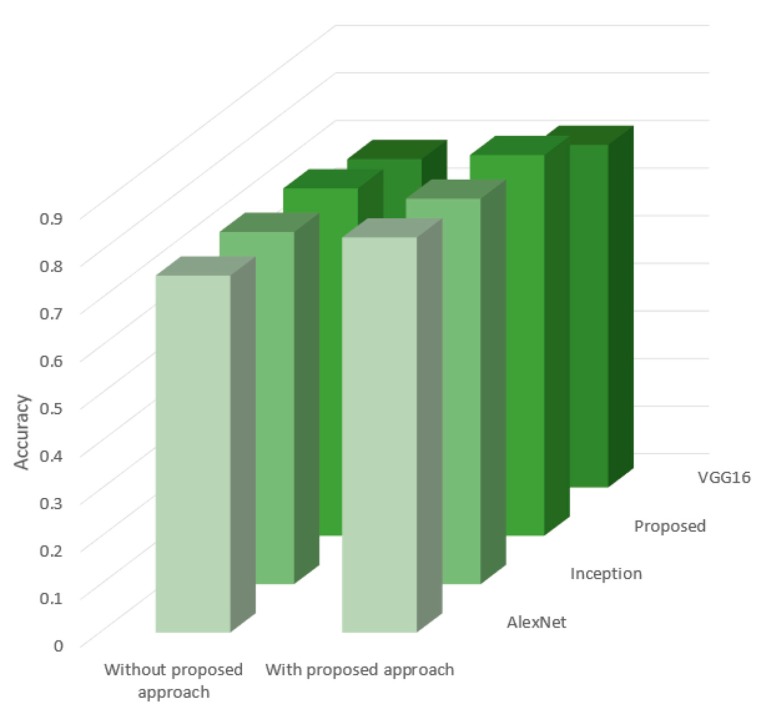
Comparison of the classic approach to image classification and hybrids.

We used other CNN architectures, including VGG16, Inception, and AlexNet, and compared the results with and without our approach, as shown in Figure 4. 

The obtained results show that all tested architectures increased classification accuracy. The average value for all architectures was around 6%, which was a good result based on small datasets (neural networks are data-hungry algorithms). However, it was noted that apart from VGG16, where the increase was close to 3%, the other architectures achieved an increase of 7%. This was a good result, which could be significant for more extensive classes in the analyzed database.

## 6. Conclusions

Image classification is a problem for which solutions are being developed all the time. In recent years, revolutionary neural networks have been developed that have enabled a huge leap forward. Unfortunately, this solution also has its problems, such as requiring a large number of samples in the database, or architecture modeling. In this paper, we focused on analyzing images of selected ships. As part of the research, we proposed a classification mechanism based on sample segments that was determined based on algorithms searching for keypoints and subsequent classification.

As part of our experiments, we performed two tests. In the first, we analyzed two classes and the results obtained showed great potential for practical applications. In the second, we used much larger sets of images of five types of ships. The proposed solution in itself showed many disadvantages, especially at the stage of determining features and assigning samples to them to train the classifier. However, we used this solution as an additional element of classification after using the classic approach, including learning transfer. As a result, we noticed that the average efficiency increased by approximately 5% in almost all cases compared to the currently used convolutional network architectures.

An analysis of the database using a feature vector, which can be treated as a bag of words, shows potential practical application, especially if the features of the objects are well described. In future research, we plan to focus on how to automatically analyze images to extract features from them, as well as automatically assign classes as an unsupervised technique.

## Figures and Tables

**Table 1:** Convolutional neural network (CNN) architecture. ReLU, rectified linear unit.

Type of Layer	Shape
Convolutional 5 × 5	(None, 96, 96, 20)
Activation: ReLU function	(None, 100, 100, 20)
MaxPooling 2 × 2	(None, 50, 50, 20)
Convolutional 5 × 5	(None, 50, 50, 50)
Activation: ReLU function	(None, 50, 50, 50)
MaxPooling 2 × 2	(None, 25, 25, 50)
Flatten	(None, 31, 250)
Dense 500	(None, 500)
Activation: ReLU function	(None, 500)
Dense 2	(None, 2)
Activation: softmax function	(None, 2)

**Table 2:** Average number of created objects using a key-search algorithm with the connection with Euclidean or river metrics. SIFT, scale-invariant feature transform; SURF, speeded up robust features; FAST, features from accelerated segment test; BRISK, binary robust invariant scalable keypoints.

	SIFT		SURF		FAST		BRISK	
	Object features	Background	Object features	Background	Object features	Background	Object features	Background
Euclidean metric	5	3	10	4	8	4	9	5
River metric	3	2	4	3	4	4	6	4

**Table 3:** Statistical coefficients for classification measurements using the selected keypoint search algorithm with Euclidean metric and CNN.

	CNN	CNN + SIFT	CNN + SURF	CNN + BRISK	CNN + FAST
Accuracy	0.78	0.814261	0.843831	0.832976	0.832347
Sensitivity	0.815476	0.903403	0.931257	0.909825	0.91635
Specificity	0.59375	0.453333	0.486842	0.534031	0.537778
Precision	0.91333	0.869979	0.881098	0.88366	0.874244
Negative predictive value	0.38	0.536842	0.634286	0.60355	0.647059
Miss rate	0.184524	0.096597	0.068743	0.090175	0.08365
Fallout	0.40625	0.546667	0.513158	0.465969	0.462222
False discovery rate	0.08667	0.130021	0.118902	0.11634	0.125756
False omission rate	0.62	0.463158	0.365714	0.39645	0.352941
F1 score	0.861635	0.886376	0.905483	0.896552	0.894802

**Table 4:** Statistical coefficients for classification measurements using the selected keypoint search algorithm with river metric and CNN.

	CNN	CNN + SIFT	CNN + SURF	CNN + BRISK	CNN + FAST
Accuracy	0.78	0.795932	0.825556	0.823859	0.820397
Sensitivity	0.815476	0.901408	0.916123	0.915133	0.923821
Specificity	0.59375	0.487113	0.521875	0.494465	0.516014
Precision	0.91333	0.837285	0.865317	0.867248	0.848889
Negative predictive value	0.38	0.627907	0.649805	0.617512	0.697115
Miss rate	0.184524	0.098592	0.083877	0.084867	0.076179
Fallout	0.40625	0.512887	0.478125	0.505535	0.483986
False discovery rate	0.08667	0.162715	0.134683	0.132752	0.151111
False omission rate	0.62	0.372093	0.350195	0.382488	0.302885
F1 score	0.861635	0.868164	0.889995	0.890547	0.884771

**Table 5:** Average classification accuracy and number of iterations in the training process.

**Iterations**	20	40	60	80	100
**Accuracy**	29%	35%	56%	61%	61%

**Table 6:** Comparison of classic CNN usage and extended usage with the proposed approach.

	Cargo	Military	Tanker	Yacht	Motorboat	Accuracy
Classic CNN	478	195	234	78	57	0.731228
Classic CNN + proposed approach	479	236	236	105	78	0.795789
